# Minimal invasive pocket technique for magnet bone implant hearing aid without fixation

**DOI:** 10.1007/s00405-019-05746-5

**Published:** 2019-12-06

**Authors:** Pierre Dolhen, Samuel Lipski, Rachid Touijar, Juliette Van Bogaert

**Affiliations:** ENT Department, CHU Tivoli, ULB, 34 avenue Max Buset, 7100 La Louvière, Belgium

**Keywords:** Bone conduction hearing aid, Bone anchored implant, Transcutaneous, Surgical technique, Minimally invasive surgery, Subperiosteal implantation

## Abstract

**Introduction:**

The BAHA (bone-anchored hearing aid) Attract is a magnetic transcutaneous bone conduction device anchored into the temporal bone. The standard surgical technique for BAHA Attract is a multi-tools time-consuming process, which requires a large cutaneous incision. The objective of this study is to describe and test the feasibility of a minimally invasive pocket (MIP) technique for Magnet Bone Implant Hearing Aid (MBIHA) with a modified magnet of BAHA Attract without fixation and without any tissue reduction. We use a 3-cm vertical skin incision and a subperiosteal pocket.

**Method:**

A study of 10 patients with conductive or mixed hearing loss who benefited from a MBIHA using the MIP technique is presented. The pure tone average (PTA) (dB) for air-conduction thresholds and the speech recognition threshold (SRT) (dB) in speech audiometry in quiet are calculated. The Entific Medical Systems (EMS) questionnaire and the postoperative clinical outcomes are realized.

**Results:**

We found a significant improvement of 33.8 dB on average for the PTA and 44.8 dB for the SRT with MBIHA at 3 months, compared with unaided situation. No implant was removed or displaced after 2 years of follow-up. The skin condition remains intact in all the cases.

**Conclusion:**

The minimally subperiosteal pocket surgical technique MIP without fixation and with tissue preservation for the MBIHA is safe, rapid and effective for patients with conductive or mixed hearing loss. It opens new perspectives of development and modify conventional concept in magnetic coupling of bone-conducted device.

**Electronic supplementary material:**

The online version of this article (10.1007/s00405-019-05746-5) contains supplementary material, which is available to authorized users.

## Introduction

Bone conduction hearing aids (BCHA) are well-accepted treatment options for audition restoration in conductive or mixed hearing loss, or in single-sided sensorineural deafness when conventional hearing aids are not tolerated or indicated, or after inefficient ossiculoplasties. Chronic otitis media, atresia of the external auditory canal and otosclerosis are the most frequent otological indications reported.

Bone-anchored hearing aid (BAHA) is a sound processor transforming sounds into vibrations, which are transferred via an osseointegrated implant to the skull bone and finally to the cochlea.

BAHA are divided into percutaneous (skin penetrating) and transcutaneous (non-skin penetrating) types. They are both fixed by an abutment to the bone.

A softband, a bone vibrator on band, or a soundarc are used for pre-operative testing or in young children who are not ready to receive a bone anchored implant because of insufficient bone thickness.

The first percutaneous osseointegrated BCHA was the BAHA with a permanent skin-penetrating abutment. Despite the good hearing outcomes [1–3], this percutaneous system can be associated with soft tissue complications such as local infection, skin overgrowth around the abutment, skin necrosis, or transcutaneous abutment loss (ranged 1.6–25%) due to infection or trauma [4–8]. In these cases, it can be difficult to replace it by another implant or device because of the damaged skin. Furthermore, some potential candidates refuse the device for esthetic concerns or fear of stigmatization.

Cochlear Bone Anchored Solutions AB (Mölnlycke, Sweden) developed the BAHA Attract in 2013. The BAHA Attract is composed of the same titanium implant (BI300) fixing the magnet (BI400) under the skin. The second external magnet is coupled magnetically with the processor. Many studies [9–13] have proven the efficiency of BAHA Attract in patients with mixed or conductive hearing loss, or single-sided sensorineural deafness has been proven in many studies and audiometric results are the same as other BCHA.

The magnetic transcutaneous anchored BAHA Attract does not have skin-penetrating abutment. It stimulates the bone by vibration of the fixed magnet through the intact skin. The advantage of transcutaneous bone conduction hearing aids is to reduce partially skin complications compared to a percutaneous abutment and need skin thinning if skin thickness is more than 6 mm. This conventional technique partially improves esthetic results, but a large 8 cm retroauricular scar is still needed.

The standard surgical technique for Attract recommended by the manufacturer is relatively complex. It implies a specific technique, multi-tools instrumentation, and constitutes a time-consuming process, requiring a large horizontal cutaneous incision ("C" shaped in retro-auricular region of 75 mm). Recommended surgery also requires the creation of a skin flap, together with bone drilling to smooth out the area for bedding the internal magnetic implant, and the use of a screw fixation system (BI300) titanium implant to attach the magnet to the bone. In case of skin exceeding 6 mm thickness, the manufacturer recommends flap thinning [14].

The major complications described (5.2%) [12, 15–17] are hematomas or seromas, wound dehiscence or infection or, more rarely, cutaneous necrosis [15, 18]. Minor complications, such as cutaneous numbness, pain, and erythema are reported in 13.1% of cases [17]. These major and minor cutaneous complications are mainly related to the creation of a large skin flap and a decrease of temporal macro- and micro-vascularization.

Therefore, we claim that the goal of developing the BAHA Attract system is only partially fulfilled the anchoring process still imposes a direct percutaneous approach or an important cutaneous flap to attach perpendicularly the magnet to the bone, which implies skin damages. To minimize those hardships, we tested an unanchored magnet implantation that minimizes vertical skin incision, adapted to the size of the magnet (2.7 cm).

To our knowledge, it is the first description of a minimally invasive pocket surgical technique (MIP) for implantation of the Magnet Bone Implant Hearing Aid (MBIHA). The MIP technique for MBIHA differs from the standard surgical technique by making a small 3 cm vertical incision distant from the implant and a subperiosteal pocket to insert the internal magnet directly on the bone. No skin flap, skin modification (thinning), bone drilling or fixation is necessary.

The objective of this study is to investigate the surgical minimally invasive pocket (MIP) aspects and to determine the audiological outcomes of MBIHA without fixation.

## Materials and methods

### Patients

This clinical study was accepted by the Hospital-Faculty Ethics Committee of Tivoli Hospital (Ref: B096201734622).

Between January 2017 and January 2018, 11 patients were implanted with a modified BAHA Attract, using the MIP surgical technique. We used the term “Magnet Bone Implant Hearing Aid” (MBIHA) in our study.

Eleven patients received MBIHA, including 4 patients with conductive unilateral hearing loss, 1 patient with conductive bilateral hearing loss, 3 patients with mixed unilateral hearing loss and 2 patients with mixed bilateral hearing loss. One patient had a single-sided sensorineural deafness and was excluded from this study to reduce bias when interpreting the audiometric results.

The mean air–bone gap (calculated as the difference between the measured hearing thresholds for air-conduction and bone-conduction) was 30 dB (SD, 12.9 dB). Chronic otitis media and otosclerosis were the otological indications for a BAHA Attract in our patients. Eight patients had a surgical history on the implanted side. The sex ratio (F/M) was 6/4 and the median age was 38 years (12–64 years). The clinical characteristics of these 10 patients are detailed in Table 1. All patients underwent a pre-operative evaluation with BAHA on soundarc for a few days.

**Table 1:** The clinical characteristics of 10 patients that received MBIHA implantations

Patient	Sex	Age	Hearing loss	Diagnosis/indication	Side of MBIHA
1	F	47	Unilateral mixte	Chronic otitis media	Left
2	M	53	Unilateral Conductive	Chronic otitis media	Left
3	F	64	Bilateral mixte	Chronic otitis media	Right
4	F	14	Unilateral conductive	Chronic otitis media	Right
5	F	62	Unilateral mixte and sensorineural controlateral	Otosclerosis	Right
6	M	47	Bilateral conductive	Chronic otitis media	Left
7	M	41	Unilateral conductive	Chronic otitis media	Left
8	F	12	Unilateral conductive	Otosclerosis	Left
9	M	60	Unilateral mixte and sensorineural contralateral	Otosclerosis	Left
10	F	61	Bilateral mixte	Otosclerosis	Left

### Implanted device

The test device was the Cochlear Baha 4 Attract system (Cochlear Bone Anchored Solutions AB, MÖlnlycke, Sweden).

The internal titanium BIM400 Implant magnet was placed underneath the skin and the Baha SP (sound processor) snaps onto the external magnet placed on the skin surface. The SP Magnet and the unfixed modified BIM400 Implant Magnet of 27 mm diameter were coupled by a magnetic retention.

The BI300 implant was not used.

The tip of the BIM400 internal magnet was removed by drilling to obtain a flat surface and the screw was removed.

External processor was the same as for conventional use and a Soft Pad was placed between the external magnet and the skin. Different magnet forces were used.

### Surgery (see video file and Fig. 1)

**Fig. 1:**
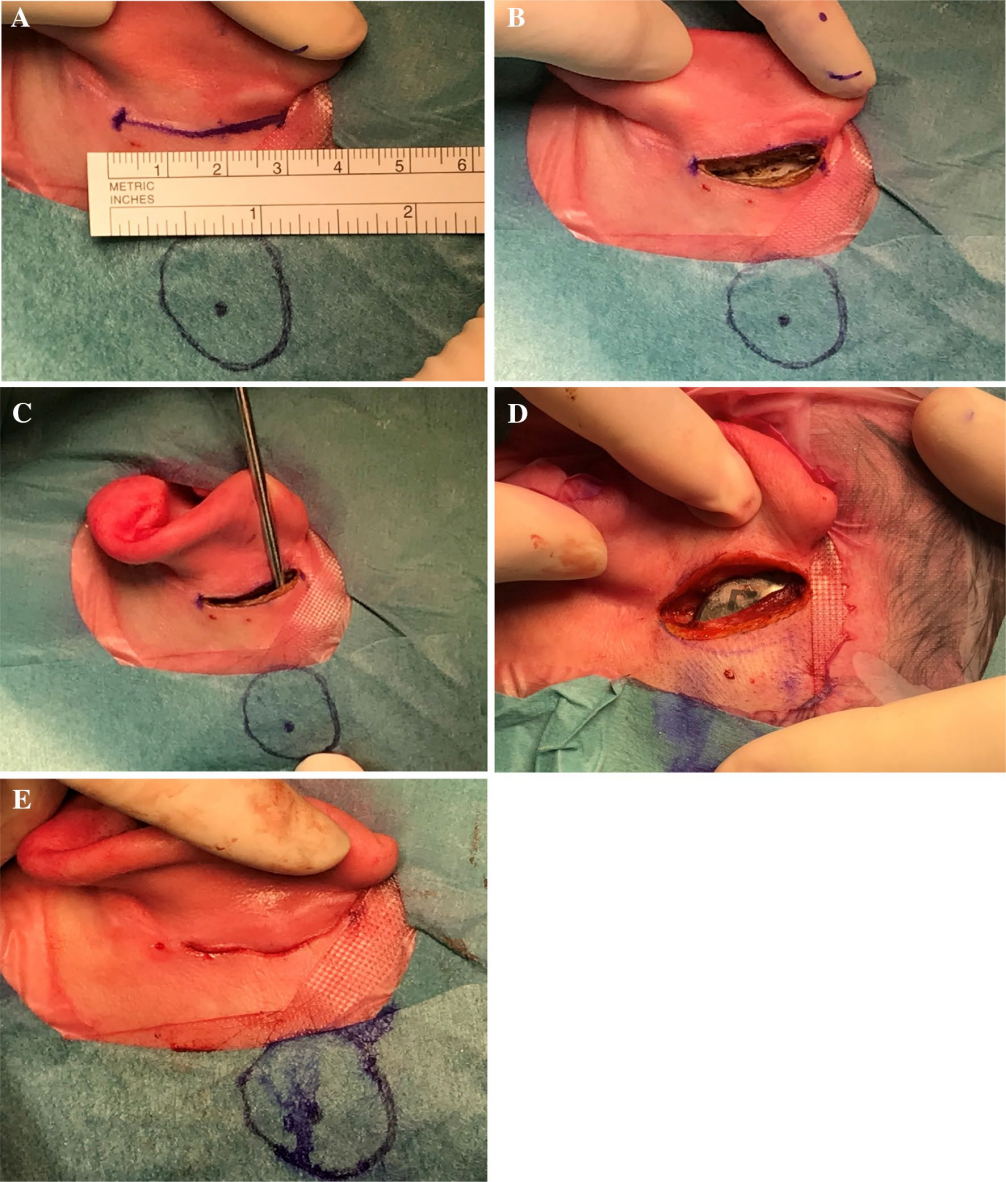
The five major surgical steps according to the MIP technique *(off label procedure)*. a Markage on the skin of the future cutaneous incision and the internal implant site projection. b Realization of a 3 cm cutaneous incision. c Creation of the subperiosteal pocket was carried out with a freer suction elevator (Storz, Germany). d Insertion of the BIM400 internal magnetic implant into the pocket. e Incision was closed in two layers

All surgeries were performed under general anesthesia.

Markage on the skin of the future cutaneous incision and the internal implant site projection was done with a pencil. Infiltration with a local anesthetic 1/100,000 or 1/200,000 xylocaine/adrenaline and 3 cm skin incision was realized.

If a pre-existing scar was present, we used the most posterior one, otherwise the incision was placed into the retroauricular fold.

The position of internal implant was usually 5–6 cm from the external ear canal.

The distance between the musculo cutaneous incision and the front edge of the magnet should be at least 2 cm.

Detachment of the subperiostal plane was carried out with a freer suction elevator (Storz, Germany) and the modified magnetic implant BIM400 was simply inserted into the pocket. Motor drill or classic instrumentation was not used.

No suture, bone or periosteal, was necessary.

Nor subcutaneoous tissue nor muscular tissue was removed even in case of thick skin.

The pocket was closed in two layers.

Compression bandage was maintained for a week.

### Audiological outcome measurements

Free-field hearing tests were performed in a soundproof audiometric chamber for unaided situation, with the BAHA on soundarc and with the MBIHA at 2, 4 and 6 weeks, 3 and 6 months after surgery.

A masking was applied to the non-tested ear.

In pure-tone audiometry, the hearing threshold for air-conduction (PTA) was calculated as the average of the thresholds measured at 0.5, 1, 2 and 4 kHz. This threshold was measured in decibel Hearing Level (dB HL or dB).

Speech audiometry in quiet was evaluated by measuring the speech recognition thresholds (SRT in dB HL) for 50% correct using dissyllabic words list (Fournier).

### Functional evaluation

Satisfaction with MBIHA was assessed in all patients using a standardized questionnaire derived from the BAHA Entific Medical Systems (EMS) Questionnaire [19, 20].

This questionnaire quantified the daily use of MBIHA in different situations and assessed the satisfaction and impact on quality of life.

### Clinical evaluation

Per and postoperative complications such as bleeding, subcutaneous hematoma, pain, skin rash, wound infection, or migration of the implant were noted.

### Statistical analysis

The Wilcoxon signed rank was used to compare the differences between paired groups (without hearing aid, with soundarc and with MBIHA) for quantitative data (average hearing thresholds in tonal and speech audiometry) (dB). Means (SD, standard deviation) were reported.

All analyses were performed using the XLSTAT software (Addinsoft, Paris, France). A *p* value less than 0.05 was accepted as significant.

## Results

### Audiological results

#### *Pure-tone audiometry*

Hearing threshold for air-conduction (dB) calculated on 500, 1000, 2000 and 4000 Hz, without hearing aids, with the BAHA on soundarc and with MBIHA at 2, 4, 6 weeks, 3 and 6 months were collected for each patient in Table 2.

**Table 2:** Pure-tone audiometry

	Without hearing aid	BAHA on soundarc	MBIHA 2 weeks	MBIHA 4 weeks	MBIHA 6 weeks	MBIHA 3 months	MBIHA 6 months
Patient 1	69	49	56	41	32	31	25
Patient 2	81	39	32	34	31	30	11
Patient 3	71	34	50	39	32	31	35
Patient 4	77	38	41	41	26	26	26
Patient 5	76	46	52	50	39	38	36
Patient 6	50	20	20	20	19	19	28
Patient 7	54	29	28	25	25	29	25
Patient 8	55	25	40	36	39	38	32
Patient 9	57	39	37	42	47	39	42
Patient 10	69	36	50	46	45	40	50
Total mean (SD)	65.9 (11)	35.5 (8.9)	40.6 (11.6)	37.4 (9.1)	33.5 (9)	32.1 (6.7)	31 (10.7)

Results of the hearing threshold for air-conduction (4PTA_AC_) (dB) calculated on 500, 1000, 2000 and 4000 Hz, preoperatively without hearing aids, with BAHA on soundarc and with MBIHA at 2, 4 and 6 weeks, and 3 and 6 months

**Fig. 2:**
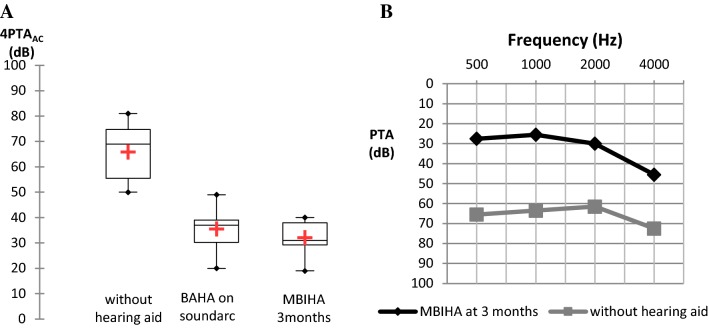
**a** Distribution of mean 4PTA_AC_ (dB) shown in a box plot for the unaided situation, for BAHA on soundarc and for MBIHA at 3 months. *N* = 10. **b** Mean pure-ton average (dB) for each specific frequency (Hz) in the situation without hearing aid and with MBIHA at 3 months. *N* = 10

Mean PTA was 65.9 dB (SD, 11 dB) without hearing aid, 35.5 dB (SD, 8.9 dB) with BAHA on soundarc and 40.6 dB (SD, 11.6 dB) with MBIHA after 2 weeks. Improvement in aided hearing performance over time was observed. Mean PTA calculated at 3 months postoperatively reached 32.1 dB (SD, 6.7 dB) (Fig. 2a).

Pure-tone audiometry showed a statistically significant improvement in PTA of 33.8 dB (SD, 12 dB; *p* = 0.005) with the test device at 3 months compared with unaided hearing situation. But no statistically significant difference in PTA compared with BAHA on soundarc was founded.

On 500 and 1000 Hz, we determined an improvement in PTA of 38 dB at 3 months. The average thresholds obtained for each specific frequency (500, 1000, 2000 and 4000 Hz) with MBIHA were, respectively, 27.2 dB, 25.5 dB, 30 dB, and 45.5 dB (Fig. 2b).

#### *Speech audiometry in quiet*

Speech recognition threshold (SRT) (dB) calculated without hearing aids, with BAHA on soundarc and with MBIHA at 2, 4 and 6 weeks, then at 3 and 6 months, were summarized for each patient in Table 3.

**Table 3:** Speech audiometry in quiet

	Without hearing aid	BAHA on soundarc	MBIHA 2 weeks	MBIHA 4 weeks	MBIHA 6 weeks	MBIHA 3 months	MBIHA 6 months
Patient 1	66	4	29	29	15	5	15
Patient 2	68	22	21	15	18	15	8
Patient 3	70	25	25	21	19	17	19
Patient 4	66	28	10	10	10	10	20
Patient 5	72	45	44	48	32	30	20
Patient 6	47	12	10	10	8	8	15
Patient 7	48	26	20	20	15	15	15
Patient 8	52	29	29	22	20	18	18
Patient 9	50	36	30	30	25	15	25
Patient 10	62	19	35	29	28	20	20
Total mean (SD)	60.1 (9.8)	24.6 (11.5)	25.3 (10.6)	23.4 (11.3)	19 (7.6)	15.3 (7)	17.5 (4.5)

Speech recognition threshold (SRT) (dB) calculated preoperatively without hearing aids, with BAHA on soundarc and with MBIHA at 2, 4 and 6 weeks, and 3 and 6 months

**Fig. 3:**
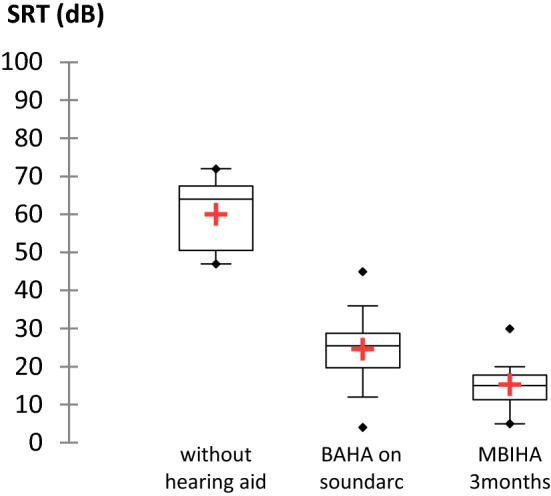
Distribution of mean SRT (dB) shown in a box plot for the unaided situation, for BAHA on soundarc and for MBIHA after 3 months. *N* = 10

Mean SRT was 60.1 dB (SD, 9.8 dB) without hearing aid, 24.6 dB (SD, 11.5 dB) with BAHA on soundarc and 25.3 dB (SD, 10.6 dB) with MBIHA at 2 weeks. The mean SRT calculated at 3 months postoperatively reached 15.3 dB (SD, 7 dB) (Fig. 3).

Speech audiometry in quiet showed a statistically significant improvement in SRT of 44.8 dB (SD, 10.1 dB; *p* = 0.005) with the test device at 3 months compared with unaided hearing situation. There was also a statistically significant difference in SRT improvement in favor of the MBIHA compared to soundarc (*p* = 0.01).

### EMS questionnaire results

**Fig. 4:**
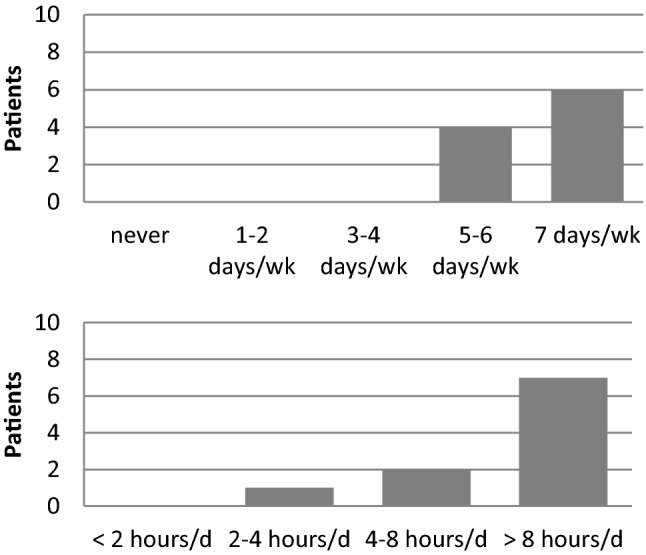
MBIHA use (days per week and hours per day)

**Fig. 5:**
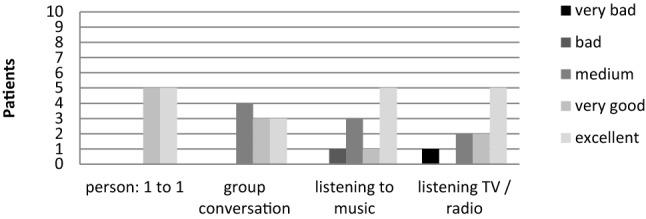
MBIHA benefit according to situation

Six patients used their Processor daily and 7 patients used it more than 8 h a day (Fig. 4). Nine patients described an improved quality of life with MBIHA. The mean overall satisfaction score was 9.1/10. In different situations, the benefit varied from moderate to excellent, with better results for discussion with only one person (Fig. 5). For the esthetical consideration, 7 patients founded that the MBIHA was discreet and not embarrassing. Eight patients reported MBIHA handling as "easy" or "very easy". At the last question, 9 patients answered that if they had to do it again, they would.

### Clinical results

No intraoperative complication was described.

Postoperatively, no hematoma or bleeding was noted. No wound infection or skin necrosis was observed.

One patient used a magnet force 3, 8 patients a force 4 and one patient a force 5.

The 14 year-old patient, with a force 4 magnet, presented a cutaneous erythema under the magnet. This erythema was resolved quickly after decreasing the magnetization force to 3.

No migration or displacement of implant during the observation period, ranging from 22 months to 33 months, was described.

**Fig. 6:**
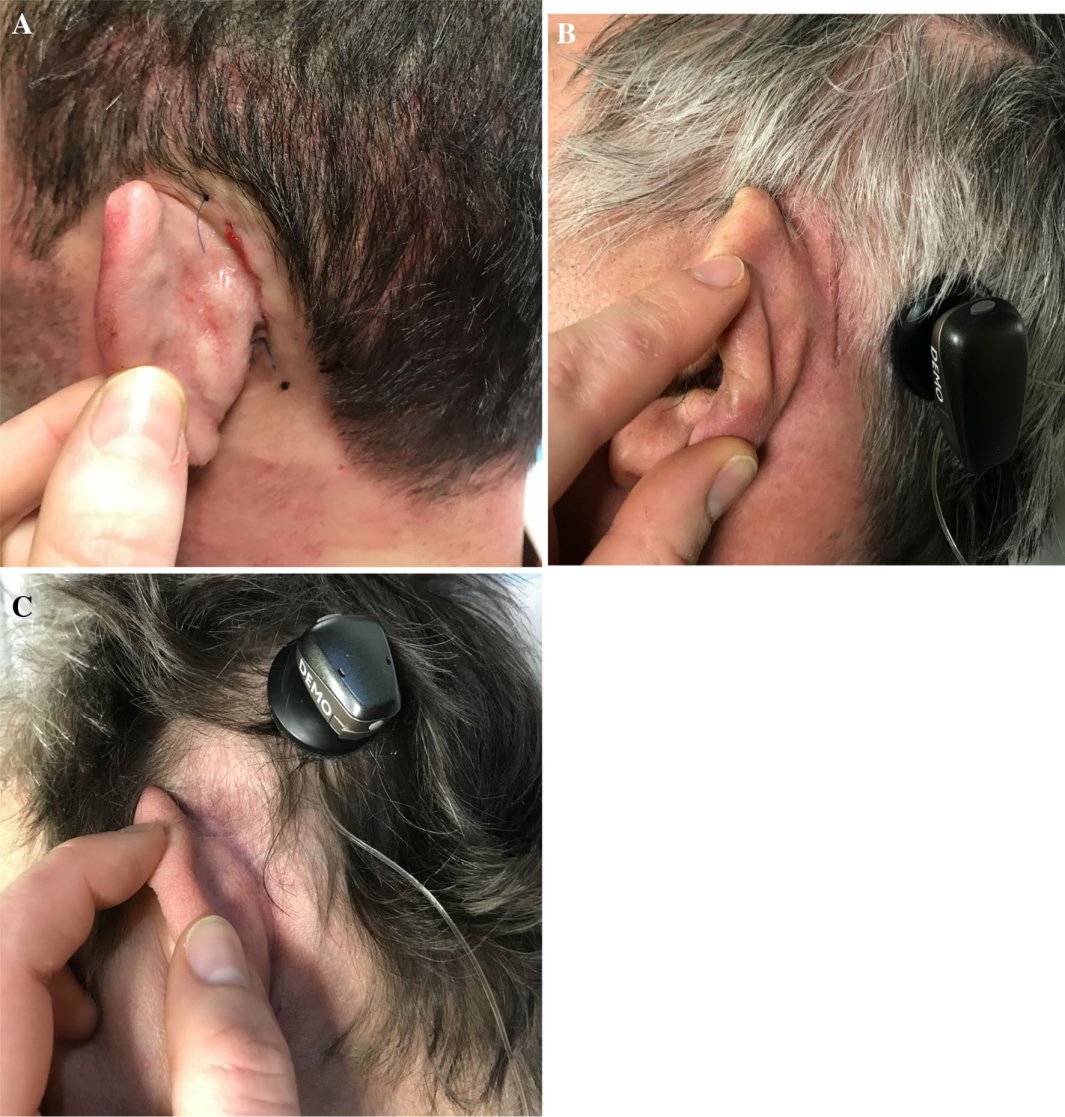
Esthetic results with MBIHA in three different patients. **a** 7 days postoperatively. **b** 14 days postoperatively. **c** 2 months postoperatively

The postoperative aspect of the retroauricular skin was illustrated in Fig. 6 and was considered as very good.

## Discussion

Our study reports a significant improvement in pure-tone and speech audiometric thresholds with the MBIHA using MIP technique compared to the unaided situation, and similar or better results compared to preoperative tests with BAHA on soundarc.

The MIP surgical technique described in our study is clearly minimally invasive. No bone drilling and no skin thinning is necessary. Because no fixation system is used, the stability of the internal magnet is obtained by a natural pericranial subperiostal pressure. Even with very thick skin a magnet force 5 is sufficient to maintain good retention.

The incision is small, vertical and is performed at least at 2 cm from the anterior edge of the magnet. It provides a security marge for direct infection through the scar tissue to the implant. In case of multi-operated patients, the most posterior pre-existing retroauricular scar can be used to avoid additional scaring. In children, preserving the retroauricular cutaneous conditions is important in case of further surgeries, specifically if cochlear implants or reconstructive surgery (for example in case of ear canal atresia) are expected in the future.

The 8 cm C-shape incision recommended for BAHA Attract includes horizontal components. Confection of cutaneous flap can potentially compromise the parieto-temporal vascularization by horizontally crossing two major arteries, the posterior branch of the superficial temporal artery and the posterior auricular artery, as described by Perenyi et al. [14]. MIP technique does not require skin flap confection; consequently, we believe that cutaneous necrosis [15, 18] is unlikely.

Major complications such as dehiscence, wound infection, cutaneous necrosis or migration are not reported in our study, after an average follow-up of 26 months. One patient developed transient cutaneous erythema in the magnet area resolved by decreasing the magnetization force of the external magnet from 4 to 3. Cooper et al. [17] described this kind of transient erythema and skin irritation in 13.1% of cases of BAHA Attract and they imputed to the pressure of the magnet on the skin and maceration. These complications are resolved quickly with the use of a smaller magnetization force [12].

The MIP technique has been already used in our hospital and by some authors for the insertion of the internal receptor-stimulator of cochlear implants (CI) [21–25]. Among these studies, some surgical techniques do not use a fixation system [22, 24] and/or do not drill a bone bed [22, 23, 25]. With this surgical technique, no case of migration or displacement of the internal receptor-stimulator has been reported [21, 22, 24, 25]. Major cutaneous complications attributed to traditional C-shaped incision was reduced [26].

In a case of surgical revision of CI, operated according to the MIP technique, a spontaneous bone bed printing was observed after 2 years of the implantation [23]. We can reasonably postulate that it will be the same process for the magnet in MBIHA.

To our knowledge, no other study was carried out using this minimally invasive surgical technique for MBIHA implantation. Actual publication still tend to find new less invasive approaches for the percutaneous abutment (without thinning [27] or with minimal skin punch incision [28]). We advocate that this innovative MBIHA implantation using MIP technique offers a new alternative with minimal invasive surgery for BCI and leads to similar bone stimulation.

Surgical time required for the implantation of MBIHA according to the MIP technique is significantly shortened (12 min on average), compared to the standard surgical technique (48 min [12]).

Since we use the softband and the soundarc on patients, we know that anchoring the magnet on the abutment is not absolutely necessary to make the skull vibrate and to stimulate efficiently the cochlea. The “no fixation” technique should be effective but still needed to be verified. Moreover, theoretically, a greater surface in contact with the skull should be more efficient to improve transmission of vibration.

Hearing results obtained with MBIHA reveal significant improvement in pure-tone and speech audiometry thresholds, compared to the situation without hearing aid, indicating an effective bone transmission trough the magnet surface.

Mean improvement in PTA with MBIHA at 3 months (33.8 dB ± 12 dB) is similar, or better than those described in the literature with BAHA Attract on patients with conductive or mixed hearing loss. These Studies revealed a mean improvement in PTA of 31 dB (Iseri et al. [9]) and 17.9 dB (Briggs et al. [11]).

Pure-tone audiometric results disclose significant improvement on all frequencies. We confirm a better improvement over 500 and 1000 Hz and a decrease in hearing performance over 4000 HZ, as described in the literature [9–11, 29]. With transcutaneous bone conduction devices, sound attenuation by the interposition of subcutaneous tissues essentially affects high frequencies but may be partially compensated by adjustment of the external sound processor.

Mean improvement in SRT with MBIHA at 3 months (44.8 dB ± 10.1 dB) is better than mean improvement in SRT measured by Iseri et al. [9] (24 dB ± 11.6 dB) with BAHA Attract on patients with conductive or mixed hearing loss.

Audiometric measurements with MBIHA are carried out at 2, 4 and 6 weeks, and 3 and 6 months postoperatively. Gradual improvement in hearing performance up to 3 months, as described by Briggs et al. [11], is founded but not statistically significant. Increase in hearing performance, essentially between 2 and 6 weeks, is explained by the gradual adaptation of the patients, progressive better contact between the magnet and the bone and the effect of the adjustments by the audioprotesist who gradually amplifies the sound. After 3 months, there is a relative stabilization of the levels of audiometric thresholds.

In our study, there is no statistically significant difference between the mean improvement in PTA with the MBIHA and the BAHA on soundarc, suggesting that preoperative soundarc tests are a good predictor of the auditory performance of the MBIHA post-operative for pure-tone audiometry [11]. However, the mean improvement in SRT measured with MBIHA at 3 months is significantly greater than with the soundarc.

The evaluation of functional results using a questionnaire confirms the reality of the daily life of patients with the MBIHA. The Entific Medical Systems (EMS) questionnaire demonstrate an overall satisfaction score of 9.1/10 with the MBIHA with an improvement in quality of life reported in 9 patients. Seventy percent of patients use their MBIHA more than 8 h per day and 100% of patients use between 5 and 7 days per week, suggesting good efficacy and comfort for most patients. Our results of the EMS questionnaire are comparable to those of the literature with BAHA [19, 20]. Powell et al. [29] used this EMS questionnaire in 6 patients with conductive or mixed hearing loss and demonstrated results with BAHA Attract that are also comparable to those in our study.

MRI realization will lead to the same problem than with the Attract and for the magnet of a CI standard unfixed magnet. A 1.5 Tesla MRI could be carried out with standard caution and bandage compression. Even if we did not remove any internal magnet until now, it may be easier to extract the internal magnet placed with the MIP technique than with the fixation technique, if a 3 Tesla MRI is absolutely needed.

Our surgical technique is interesting as it potentially addresses some of the concerns with BAHA implantation; most specifically the need for adequate calvarias thickness and solidity to support osseointegration of the implant fixture as well as limited impact on the retroauricular skin for a similar efficiency.

It is a very simple “single tool” technique giving comparable result to those obtained with the soundarc, the BAHA Attract system or the percutaneous abutment. We demonstrate that rigid fixation is not needed to obtain excellent audiometric results.

Finally, magnetic coupling of a BCI processor becomes unspecific to one producer and should stimulate development of a larger number of processor products, which address patient concern like aesthetic, price and performance. Minimally aspect of the procedure and future work on MRI compatibility of the magnet (neutral comportment if a MRI is necessary) promotes the MBIHA using MIP technique, particularly for a pediatric population or patients with poor bone condition, like Lobstein disease or post radiotherapy.

## Conclusion

The minimally invasive pocket technique described for the first time in our study for the Magnet Bone Implant Hearing Aid possesses several advantages over the standard technique for BAHA Attract:

- Easier to perform
- Minimal instrumentation required
- Minimal 3 cm vertical incision
- Faster healing
- Early fitting possible
- Better esthetic result
- Time saving

The size, the orientation of the skin incision, any skin thinning and the subperiosteal detachment are important factors to preserve the cutaneous perfusion of the retroauricular tissue on and near the implant, allowing to reduce complications. Our results confirm the minimally invasive aspects of the MIP technique without implant migration. The MIP technique preserves the retroauricular skin. It must than be recommended for BCI, especially in pediatric population with thin bone and in patients with poor quality bone. The audiological results obtained with MBIHA without fixation and implanted using the MIP surgical technique, in patients with conductive or mixed hearing loss, are comparable to the results described with the standard surgical technique.

## Electronic supplementary material

Below is the link to the electronic supplementary material.

Supplementary file1 (MP4 137079 kb)

## Abbreviations

BAHA: Bone-anchored hearing aid
BCHA: Bone conduction hearing aid
dB HL or dB: Decibel hearing level
CI: Cochlear implant
EMS: Entific medical systems
Hz: Hertz
MBIHA: Magnet bone implant hearing aid
MIP: Minimal invasive pocket
PTA: Pure-tone average
SD: Standard deviation
SRT: Speech recognition threshold
